# Evaluation of Predation Capability of Periodontopathogens Bacteria by Bdellovibrio Bacteriovorus HD100. An in Vitro Study

**DOI:** 10.3390/ma12122008

**Published:** 2019-06-23

**Authors:** Romeo Patini, Paola Cattani, Simona Marchetti, Gaetano Isola, Gianluca Quaranta, Patrizia Gallenzi

**Affiliations:** 1 Fondazione Policlinico Universitario A. Gemelli IRCCS, Institute of Dentistry and Maxillofacial Surgery, Università Cattolica del Sacro Cuore, 00168 Roma, Italy; romeo.patini@unicatt.it (R.P.); patrizia.gallenzi@unicatt.it (P.G.); 2 Fondazione Policlinico Universitario A. Gemelli IRCCS, Institute of Microbiology and Virology, Università Cattolica del Sacro Cuore, 00168 Roma, Italy; paola.cattani@unicatt.it (P.C.); simona.marchetti@policlinicogemelli.it (S.M.); 3 Department of General Surgery and Surgical-Medical Specialties, Via Plebiscito 628, University of Catania, 95124 Catania, Italy; gaetano.isola@unict.it

**Keywords:** Local antimicrobial therapy, microbiology, periodontal medicine, periodontitis, plaque control

## Abstract

Treatment options against periodontitis attempt to completely remove oral microbiota even if several species in dental plaque demonstrate protective features. Predatory bacteria that selectively predate solely on Gram-negative bacteria might be a viable therapeutic alternative. Therefore, the aim of this study is to in vitro evaluate the susceptibility of some oral pathogens to predation by *B. bacteriovorus* HD100 in liquid suspension. Cultures of prey cell were prepared in brain heart infusion broth (BHI) broth incubating overnight at the appropriate conditions for each organism to reach log phase of growth. Predatory activity was assessed by measuring optical density at 600 nm after 12, 24, 48 and 72 hours. Statistical analysis was performed using the Mann–Whitney U test and p values less than 0.05 were considered statistically significant. The study demonstrated that *B. bacteriovorus* is able to predate on aerobic species and on microaerophilic ones (p < 0.05) but also that its predatory capacity is strongly compromised by the conditions of anaerobiosis. *B. bacteriovorus*, in fact, was unable to predate the anaerobic species involved in the present study (*F. nucleatum* and *P. gingivalis*). The findings of the study suggest that *B. bacteriovorus* is able to tolerate microaerophilic conditions and that in anaerobiosis it cannot exert its predatory capacity. Such evidence could lead to its use as an agent to prevent recolonization of the periodontal pocket following therapy. Further studies are needed to investigate the activity of *B. bacteriovorus* against recently recognized periodontopathogens, alone or organized in biofilms of multi-species communities.

## 1. Introduction

Periodontal disease is a multifactorial infection in which a complex of bacterial species interact with host tissues and, if left untreated, cause the destruction of periodontal structures.

According to recent reports, the severe form of periodontitis is the sixth most prevalent disease worldwide; it has an overall prevalence of 11.2% and around 743 million people are affected. From 1990 to 2010 its global burden increased by 57.3% [1,2]. The bacteria most frequently isolated from patients affected by periodontal diseases mainly belong to the Gram-negative category, such as: *Aggregatibacter actinomycetemcomitans, Eikenella corrodens, Fusobacterium nucleatum, Prevotella intermedia, Porphyromonas gingivalis* and *Tannerella forsythia* and their crucial role in the etiopathogenesis of periodontal diseases is widely known [3]. For this reason, numerous efforts have been made to effectively control the intra-oral bacterial load of the known pathogenic species.

Furthermore, it has recently been shown that new bacterial species could have a potential pathogen role for periodontal disease etiology and progression [4] and that some systemic diseases and syndromes are related to an increase on the activity of the cells of the immune system and a worsening of periodontal clinical conditions [5,6,7]. 

The suspected periodontal pathogens are not frequently found in extraoral infections. Nonetheless it has been demonstrated that some microorganisms like *Synergistes* spp. and *Peptostreptococcus* spp. may also act as an opportunistic pathogen if they succeed in infecting other types of mucosa or skin tissues [8]. Among these *Parvimonas micra* has been isolated into polymicrobial infections connected to pleural empyema [9] and septic arthritis [10].

Even if Roberts et al. demonstrated that some bacteria could be beneficial for the periodontium and that they should not be eradicated [11], currently used therapeutic strategies against periodontal diseases basically aim at the complete plaque removal by physical or chemical means leaving recolonization up to chance. 

The difficulty in removing oral plaque or biofilms by conventional therapies led researchers to examine other alternative methods for biofilm control, such as biological control agents. One biological agent that might be used to control pathogenic bacteria is the predatory prokaryotes from the genus *Bdellovibrio*. The most studied strain of the *Bdellovibrio* and like organisms (BALOs) group is *Bdellovibrio bacteriovorus*. *B. bacteriovorus* is a delta-proteobacterium with high motility that predate solely on Gram-negative bacteria [12,13]. The predatory selectivity of *B. bacteriovorus* against Gram-negative bacteria is due to its peculiar predation mechanism. Thanks to the flagellum of which it is endowed, in fact, *B. bacteriovorus* succeeds in invading the prey cells penetrating through their outer membrane. In the periplasmic space, the predator begins its replicative cycle that will end with the lysis of the host cell [12]. Previous research has demonstrated that *B. bacteriovorus* HD100 had the widest prey spectrum and was able even to reduce *A. actinomycetemcomitans* biofilm formation [14]. 

It has been shown that periodontal diseases are sustained by a dense series of Gram-negative bacterial species [4] and that the species that make up the protective part of the oral microbiota almost completely belong to the Gram-positive species. In light of this, the use of a selective biological agent in the control of such infections may represent a valid alternative or, at least, integration to antibiotic therapies that cause an almost complete eradication of the oral cavity microbiota. *B. bacteriovorus*, in fact, only predate on Gram-negative bacteria leaving the Gram-positive part in the oral cavity unaffected [14].

There is currently no data on the predatory capacity of *B. bacteriovorus* HD100 against potential periodontopathogenic bacteria under different oxygen conditions.

Therefore, the aim of this study was to in vitro evaluate the susceptibility of some oral pathogens (*Escherichia coli*, *Eikenella corrodens*, *Fusobacterium nucleatum*, *Aggregatibacter actinomycetemcomitans* and *Porphyromonas gingivalis*) to predation by *B. bacteriovorus* HD100 in liquid suspension according to their type of cellular respiration.

## 2. Materials and Methods

### 2.1. Bacteria Strains and Growth Conditions

The periodontopathogens bacteria selected as preys for the study were *Escherichia coli* ATCC-11229, *Eikenella corrodens* ATCC-23834, *Aggregatibacter actinomycetemcomitans* ATCC-43717, *Porphyromonas gingivalis* ATCC-33277 and *Fusobacterium nucleatum* ATCC 25586. They were acquired by microorganism collection LGC STANDARD S.r.l (Milan, Italy) and arrived at laboratory in lyophilized form. *Bdellovibrio bacteriovorus* ATCC 15356 strain HD100 was chosen as predator. 

Two growth media were used for the growth of periodontopathogenic strains: TSA agar for aerobic species that were incubated overnight at 37 °C for 24 hours and Schaedler agar for anaerobes (Becton-Dickinson Heidelberg/Germany), incubated overnight for 48–72 hours at 37 °C in anaerobiosis guaranteed by anaerobic hood. The anaerobic atmosphere was reproduced using pouches AnaeroGen (Thermo Scientific, Waltham, MA, USA) whereas microaerophilic conditions with CampyGen pouches (Thermo Scientific, Waltham, MA, USA). Bacterial concentration was adjusted by measuring optical density at 600 nm to reach the concentration of 1 × 10^8^ CFU/ml. Two or three colonies obtained by each strain were then transferred to 10 ml Brain Heart Infusion broth (BHI) (Thermo Scientific, Waltham, MA, USA).

### 2.2. Preparation of B. Bacteriovorus Suspensions

Colonies of freshly grown *E. coli* culture was prepared in 10–15 mL of BHI broth. Lyophilized *B. bacteriovorus* was rehydrated using 0.5 mL of this culture, added into the remaining host culture and incubated aerobically at 30 °C with rotary shaking until the co-culture became clear (stock lysate). To harvest the predators, 2 mL of host cell culture (1 × 10^8^ CFU/ml) were inoculated with 2 mL of stock lysate in 20 mL of BHI broth to obtain a fresh lysate after 18–24 h of aerobic incubation at 30 °C under continuous shaking. Before harvesting the predatory bacteria, liquid cultures were evaluated with phase contrast microscopy. *B. bacteriovorus* cells were separated from the remaining prey by filtrations through a 0.45-µm pore-size filter (Sartorius, Goettingen, Germany) to remove residual prey and cell debris (filtered lysate). One drop of filtered preparation was plated on TSA agar plate and incubated for 24 h to verify no prey grown. The optimal concentration of pure filtered predator, for predation assay, was evaluated by the reduction in host cell viability (CFU enumeration) in repeated previous experiments at the same conditions.

### 2.3. Prey Range Assay

To evaluate the predatory ability of *B. bacteriovorus* on selected pathogens, cultures of prey cell were prepared in BHI broth incubating overnight at the appropriate conditions for each organism to reach log phase of growth. *Eikenella corrodens* and *Escherichia coli* were incubated for 24 h at 37 °C with shaking and *Aggregatibacter actinomycetemcomitans* was incubated for 24 h at 37 °C in microaerophilic conditions. For anaerobic species incubation at the same temperature for 48 h under anaerobic conditions was performed. Two milliliter of filtered *B. bacteriovorus* (1 × 10^8^ PFU/ml) were added to 20 mL of BHI prey cell cultures in their log phase of growth, then distributed in 96 well plates, in triplicate, and incubated in aerobiosis for *Eikenella corrodens* and *Escherichia coli*, in microaerophilic conditions for *Aggregatibacter actinomycetemcomitans* and in anaerobiosis for *F. nucleatum* and *P. gingivalis* for up to 72 h, at 37 °C on a rotary shaker as described below:

- I Group: Prey without predator as positive control;
- II Group: Prey with predator;
- III Group: Predator without prey as negative control.

Predatory activity was assessed by measuring optical density at 600 nm (Spectrophotometer EL808nBioTEK, PBI international) after 12, 24, 48 and 72 hours. The ability of predator was also confirmed by the reduction of host cell viability, measured by CFU enumeration, compared to the predator free control. 

### 2.4. Statistical Analysis

The Mann–Whitney U test was employed to assess the different susceptibility of oral pathogens predation by *B. bacteriovorus*. Statistical analysis was performed using Med Calc software version 8.0 (MedCalc Software, Ostend, Belgium). P values less than 0.05 were considered statistically significant. The sample size was calculated from a power analysis based on the results of a previous study [14] to detect at least a reduction of 0.5 OD600 of turbidity with a standard deviation of 0.1. The α and β values were set as 0.05 and 0.90, respectively, and the sample size was calculated to be 80 samples for each prey.

## 3. Results

**Figure 1:**
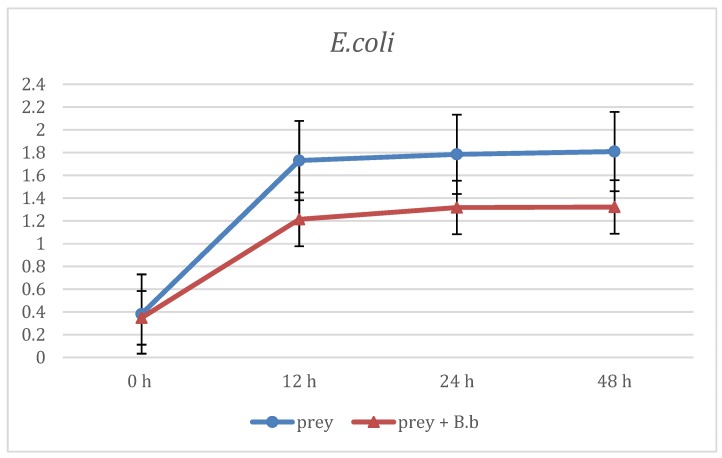
Predation activity of *B. bacteriovorus* against *E. coli*.

**Figure 2:**
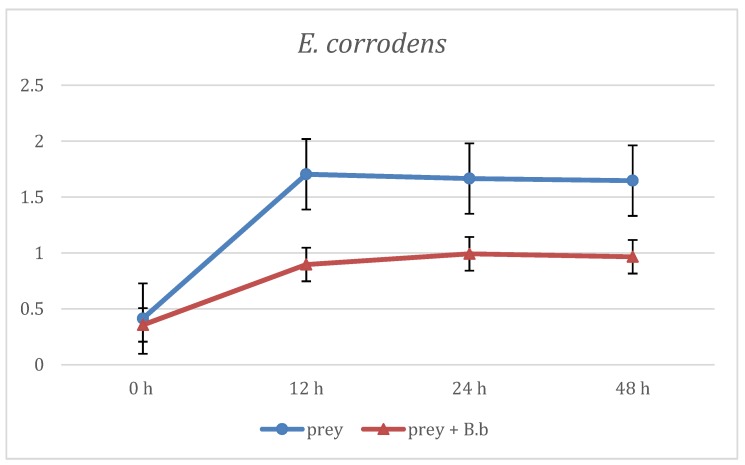
Predation activity of *B. bacteriovorus* against *E. corrodens*.

**Figure 3:**
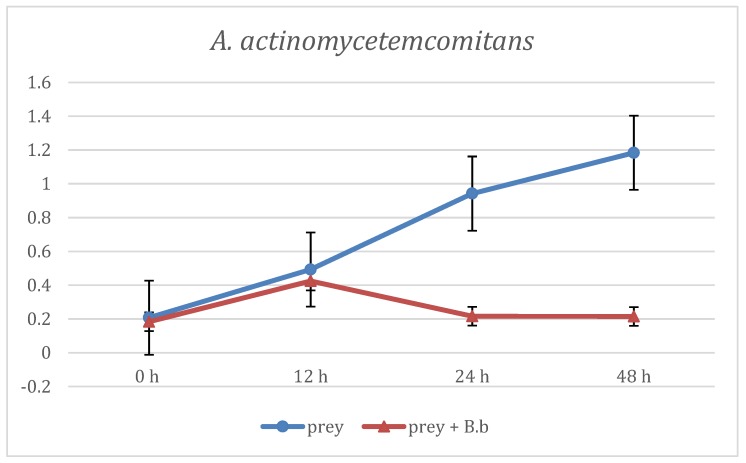
Predation activity of *B. bacteriovorus* against *A. actinomycetemcomitans*.

**Figure 4:**
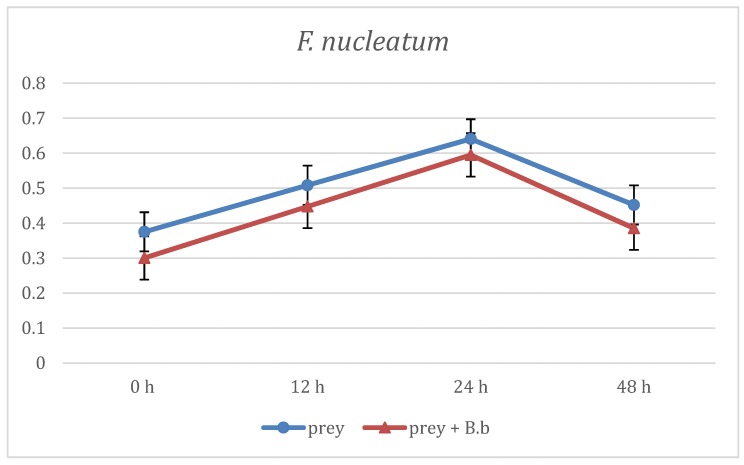
Predation activity of *B. bacteriovorus* against *F. nucleatum*.

**Figure 5:**
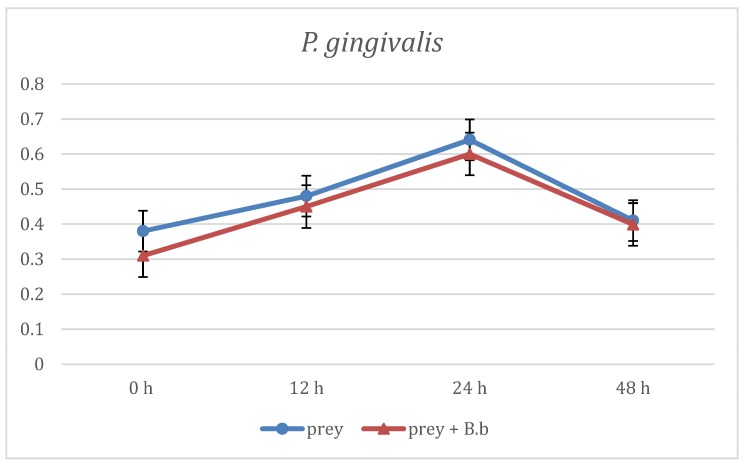
Predation activity of *B. bacteriovorus* against *P. gingivalis*.

**Figure 6:**
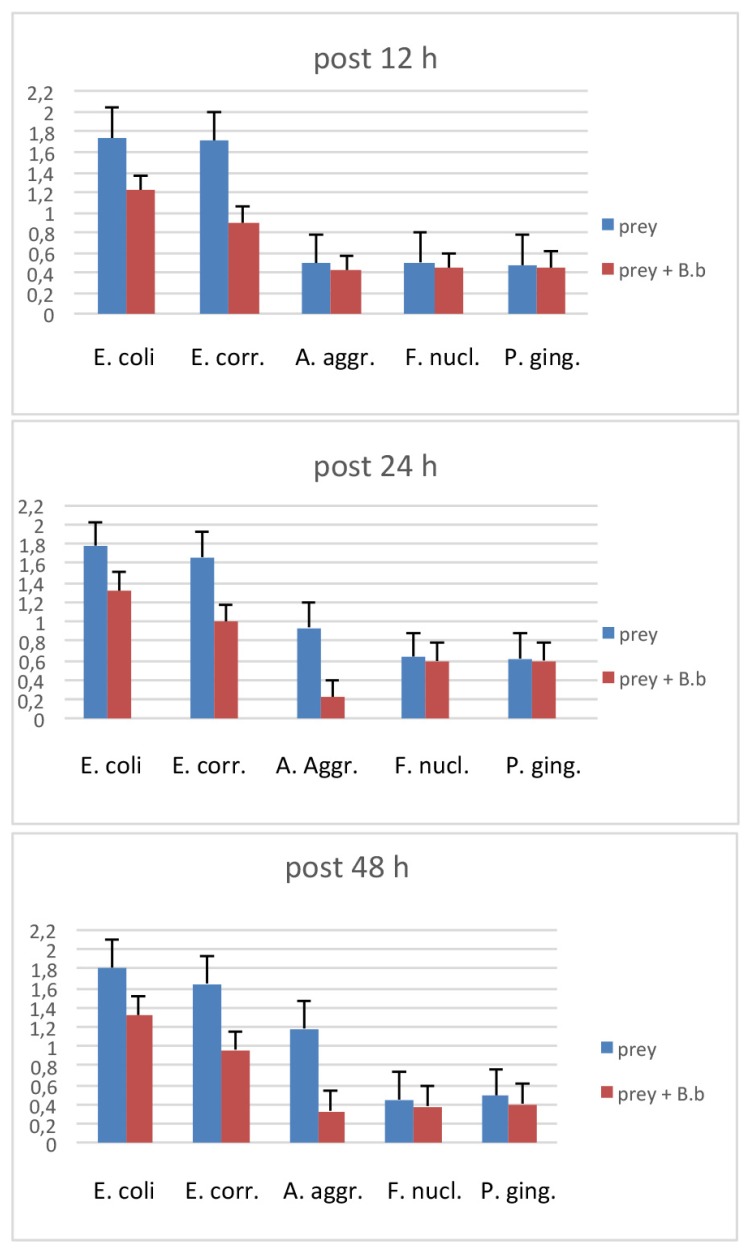
Difference of bacterial growth rate between positive controls and co-cultures at 12 h, 24 h and 48 h.

The first evaluation of *B. bacteriovorus* predation was carried out on two aerobic species: *E. coli* and *E. corrodens*, grown on BHI broth. As reported in Figure 1 at the first time point (12 h) already, the curve showing the predation activity of *B. bacteriovorus* on *E.coli* highlighted a significant (p < 0.05) reduction of OD mean values (OD600 = 1.21 ± 0.88) when compared to the prey alone (OD600 = 1.73 ± 0.02). Such trend of reduction was observed also at the two subsequent time points, respectively at 24 h (OD600 = 1.31 ± 0.01 vs 1.78 ± 0.02) and at 48 h (OD600 = 1.32 ± 0.06 vs 1.8 ± 0.06). Regarding on *B. bacteriovorus* predatory activity against *E. corrodens* (Figure 2) the pick of growth of the positive control was reached at 12 h (OD600 = 1.8 ± 0.06) and it was maintained for the entire observation period; the presence of predator on prey culture caused a marked (p < 0.05) reduction of turbidity in all time point (12 h: OD600 = 0.89 ± 0.29; 24 h: OD600 = 0.99 ± 0.1; 48 h: OD600 = 0.96 ± 0.05). In order to evaluate *B. bacteriovorus* activity against microaerophilic periodontopathogens predatory assays were carried out on *A. actinomycetemcomitans*. No significant differences (p = 0.08) were observed during the first 12 h of observation between the positive controls and the co-cultures (0 h: OD600 = 0.20 ± 0.02 vs. OD600= 0.18 ± 0.02 and 12 h: OD600 = 0.49 ± 0.08 vs. OD600 = 0.42 ± 0.03). At 24 h and 48 h time points a significant (p < 0.05) remarkable difference in terms of turbidity was observed (24 h: ΔOD600 = 0.72; 48 h: ΔOD600 = 0.96) (Figure 3). Regarding on anaerobic species, the authors decided to evaluate the predatory activity of *B. bacteriovorus* against *F. nucleatum* and *P. gingivalis*. Active predation towards such bacteria was not detected since OD values in positive control and in co-culture were quite similar (Figure 4 and Figure 5). The differences of bacterial growth rate between positive controls and co-cultures were described in bar graphs in which OD values were reported in percentages for every time point (Figure 6). At 12 h the reduction of growth of *E.coli, E. corrodens, A. actinomycetemcomitans, F. nucleatum* and *P. gingivalis* was respectively: 30.06 %, 47.6 %, 14.29 %, 11.76 % and 6.25 %. At 24 h and 48 h time points all growth rate values remained stable except for *A. actinomycetemcomitans* whose decrease was assessed at 76.6% and 81.36%, respectively.

## 4. Discussion

With the aim of finding an alternative, less invasive, treatment strategy against periodontal disease, the present work successfully demonstrated the ability of *B. bacteriovorus* HD100 to predate some of the well-known periodontopathogens bacteria. BALOs were accidentally discovered in the 1960s as constituents of soil, rivers, oceans, sewage, and even of human intestine [15]. All bacteria belonging to the BALOs group are unable to infect eukaryotic cells [16] and *Bdellovibrio* lipopolysaccharide is not able to induce a strong immunological response [17]. BALOs showed the ability to predate Gram-negative bacteria so they were soon considered as potential biological agents for the control of human infections sustained by such type of bacteria [14]. The present study demonstrated that *B. bacteriovorus* is able to predate on aerobic species (*E. coli* and *E. corrodens*) and on microaerophilic ones (*A. actinomycetemcomitans*) as previously found by Van Essche et al. [18] but it is the first to highlight that its predatory capacity is strongly compromised by the conditions of anaerobiosis. *B. bacteriovorus*, in fact, was absolutely unable to predate the anaerobic species involved in the present study (*F. nucleatum* and *P. gingivalis*). Regarding this aspect, any comparison with the previous scientific literature is difficult to do because previous studies have been conducted under aerobic conditions to promote the development of the *B. bacteriovorus* life cycle [18]. Since the genome analysis of the *B. bacteriovorus* HD100 allows us to speculate a number of adaptations to make anaerobic respiration possible [18], in this study, the experiments with anaerobic bacteria were conducted in anaerobiosis to simulate the environment of the deepest part of the gingival sulcus and to avoid the bias of the false positive linked to the premature death of the prey. However, our findings suggest that *B. bacteriovorus* HD100 was able to tolerate microaerophilic conditions and, only for limited periods, also anaerobiosis. In fact, in the co-culture of *B. bacteriovorus* with *F. nucleatum and P. gingivalis* the anaerobic condition strongly interfered with predator’s growth, severely affecting its predatory ability. This evidence had been previously demonstrated only on the *B. bacteriovorus* 109J strain [19]. The bacteria used as prey were chosen by the authors among those considered in close relationship with periodontal diseases. [3,4] The susceptibility of *A. actinomycetemcomitans* to *B. bacteriovorus* highlighted by this work opens a window on the potential use of BALOs in the treatment of the forms of periodontal disease supported by a specific pathogenic flora [4,20]. Nowadays the treatment of such forms, in fact, involves a causal therapy that eliminates bacterial plaque, possibly surgical treatment, and in cases of aggressive periodontitis; the use of broad-spectrum antibiotics that can alter the composition of the oral microbiota, also killing bacteria with a protective role [7]. Considering the strong similarity between the bacterial flora that causes periodontitis and the one that causes peri-implantitis [21], it is reasonable to hypothesize a potential use of *B. bacteriovorus* in the treatment of infectious diseases involving dental implants. Some limitations of this study must also be reported. According to the results of the experiments, *B. bacteriovorus* was unable to predate the obligate anaerobic bacteria that are commonly considered the most active in periodontal destruction. Nevertheless, such limitation, combined with the predatory activity demonstrated against microaerophilic and aerobic species, could lead to the use of *B. bacteriovorus* as an agent to prevent recolonization of the periodontal pocket following periodontal therapy and of dysplastic diseases connected with tooth impaction [14,22,23,24]. Another limitation could be linked to the fact that predatory activity was assessed through the use of optical density at 600 nm instead of quantitative polymerase chain reaction (qPCR). The use of qPCR was deliberately avoided since it analyzes specific DNA sequences present in a biological sample without distinguishing between DNA from viable and killed bacterial prey cells by requiring careful evaluations to establish the predatory activity of *B. bacteriovorus*.

## 5. Conclusions

In this study, the authors demonstrated the potential use of *B. bacteriovorus* HD100 in controlling oral pathogens in vitro. Considering the intrinsic limits of in vitro experiments, further studies are needed to investigate the predatory activity of *B. bacteriovorus* against recently recognized periodontopathogens [4], alone or organized in multi-species communities, under different oxygen conditions and in presence of human saliva and/or other environmental factors that might influence predator-prey relationship. The correct evaluation of such an anti-infective agent, in fact, should be a clinical trial in which it is administered in patients after periodontal causal therapy.
